# Neurobiology of Cancer: Introduction of New Drugs in the Treatment and Prevention of Cancer

**DOI:** 10.3390/ijms22116115

**Published:** 2021-06-06

**Authors:** Boris Mravec

**Affiliations:** 1 Institute of Physiology, Faculty of Medicine, Comenius University in Bratislava, 813 72 Bratislava, Slovakia; boris.mravec@fmed.uniba.sk; Tel.: +421-(2)-59357527; Fax: +421-(2)-59357601; 2 Biomedical Research Center, Institute of Experimental Endocrinology, Slovak Academy of Sciences, 845 05 Bratislava, Slovakia

**Keywords:** β-blockers, adrenergic, antibodies against nerve growth factor, aspirin, cancer, electroceuticals, local anesthetics, metformin, neurobiology of cancer, propranolol

## Abstract

Research on the neurobiology of cancer, which lies at the border of neuroscience and oncology, has elucidated the mechanisms and pathways that enable the nervous system to modulate processes associated with cancer initiation and progression. This research has also shown that several drugs which modulate interactions between the nervous system and the tumor micro- and macroenvironments significantly reduced the progression of cancer in animal models. Encouraging results were also provided by prospective clinical trials investigating the effect of drugs that reduce adrenergic signaling on the course of cancer in oncological patients. Moreover, it has been shown that reducing adrenergic signaling might also reduce the incidence of cancer in animal models, as well as in humans. However, even if many experimental and clinical findings have confirmed the preventive and therapeutic potential of drugs that reduce the stimulatory effect of the nervous system on processes related to cancer initiation and progression, several questions remain unanswered. Therefore, the aim of this review is to critically evaluate the efficiency of these drugs and to discuss questions that need to be answered before their introduction into conventional cancer treatment and prevention.

## 1. Introduction

Data accumulated in the last few decades have clearly shown that the nervous system plays a significant role in cancer initiation and progression. These data are based on combined neuroscientific and oncological research that has created the basis for a new scientific concept, the so-called neurobiology of cancer [1]. This research elucidates the mechanisms and pathways participating in interactions between the nervous system and cancer.

Experimental and clinical studies have shown that drugs that modulate the transmission of signals between the nervous system and the tumor micro- and macroenvironments efficiently suppress cancer initiation and progression. These data indicate that pharmacological approaches based on findings of the neurobiology of cancer might be utilized in the treatment of cancer patients as well as in cancer prevention for individuals with an increased cancer risk. However, there are several questions that need to be answered before introducing these new treatments into oncology. The following sections are focused on drugs that are approved for the treatment of non-cancerous diseases, as well as experimental drugs, that, via modulation of nervous system-related signaling and functions, could affect carcinogenesis and cancer growth.

## 2. Propranolol

An important role in modulating carcinogenesis and cancer progression is played by the autonomic division of the nervous system, especially the sympathoadrenal system [2,3]. The sympathoadrenal system consists of sympathetic nerves releasing norepinephrine into innervated tissues and the adrenal medulla, releasing into the bloodstream epinephrine and, in lesser amounts, norepinephrine [4]. Epinephrine and norepinephrine exert their effects via binding on adrenergic receptors expressed by target cells. These receptors are divided into α and β types and further subdivided into several subtypes [5]. Sympathoadrenal system signaling mediated via the β_2_ subtype of adrenergic receptors plays a central role in processes related to cancer development and progression [2,6]. However, it is necessary to note that activation of other subtypes of α and β adrenergic receptors might also affect cancer [7,8,9,10,11,12]. Epinephrine and norepinephrine might affect cancer initiation and progression via activation of one or more of the intracytoplasmic signaling cascades implicated in cancer, such as Ras/MAPK, PI3K/Akt, and JAK/STAT [2,13].

**Figure 1:**
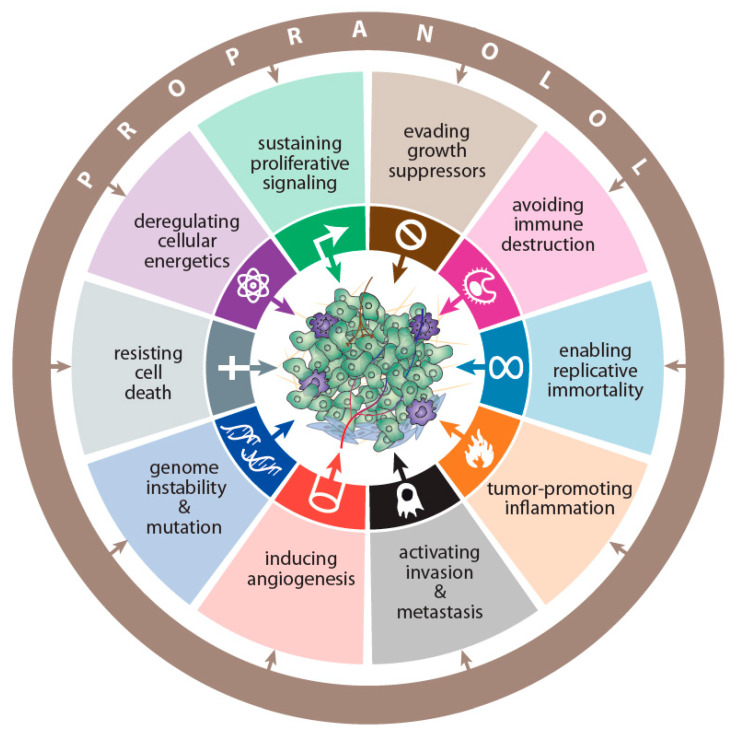
Experimental and clinical studies have shown that propranolol affects all hallmarks of cancer defined by Hanahan and Weinberg [52]. Even if these studies created a basis for potentially employing propranolol in cancer treatment and prevention, further studies are necessary to determine its efficacy in various human cancers.

It has been shown that β-adrenergic signaling affects DNA mutations and repair in animal models of cancer and in in vitro experiments on human cancer cells [14,15,16,17]. It has also been shown to activate oncogene-related signaling [18,19,20], sensitize cells to carcinogens [21,22], increase stemness [23], promote cancer-related inflammation [24], increase the proliferation of cancer cells [25,26,27], participate in the altered energetics of cancer cells [13,28], promote neovascularization of cancer tissue and increase the activity of matrix metalloproteinases [29,30,31,32], rearrange lymphatic vessels within and around tumor tissue [33], increase both the stiffness [34] and migration of cancer cells [26,35,36], and potentiate the development of metastases [37,38,39,40]. The majority of these effects might be significantly reduced by antagonists of β_2_-adrenergic receptors. The most frequently used antagonist of the β_2_-adrenergic receptor in research on cancer neurobiology is propranolol (Figure 1).

Propranolol is a non-selective β blocker [41]. However, it is necessary to note that even if propranolol blocks both β_1_- and β_2_-adrenergic receptors, experiments comparing the effects of propranolol treatment with the effects of selective antagonists of β_1_-adrenergic receptors clearly showed that the effects of propranolol on cancer induction and progression are mediated by the reduction of β_2_-adrenergic signaling. Effects similar to those of propranolol might also be induced by the administration of a selective antagonist of β_2_-adrenergic receptors, such as the experimental compound ICI-118,551.

There are several reasons that propranolol is the most often used β blocker in the investigation of neurobiology of cancer: (a) it was the first β blocker effectively used in the treatment of coronary artery disease and hypertension and therefore has been used in clinical practice for a long time [41]; (b) its pharmacological properties have been described in detail [42]; (c) there have been several retrospective studies involving patients treated with propranolol that have investigated the effect of β blockers on cancer incidence and progression [43,44,45,46,47,48,49,50,51]; (d) propranolol is readily available, inexpensive, and easily applicable in animal experiments; (e) as a clinically approved drug, off-label propranolol use by cancer patients in clinical trials is highly likely to be considered acceptable by the approving authorities of these studies.

### 2.1. Propranolol and Cancer Incidence

The reduction of cancer incidence by propranolol has been demonstrated in both animal and clinical studies. In animal models, propranolol reduces the incidence of several cancer types, including prostate, pancreas, and tongue [7,53,54]. Data from our laboratory have shown that propranolol treatment also reduced the development of chemically induced mammary carcinoma in rats [55]. Similar effects were also found in retrospective clinical studies showing that long-term treatment of non-cancerous diseases by propranolol before cancer diagnosis reduced the incidence of head and neck, esophagus, stomach, liver, colon, and prostate cancers [43,56,57,58]. However, it is necessary to note that published data from retrospective studies showing a reduction in cancer incidence in patients treated by propranolol might also reflect a suppressive effect of this β blocker on already present dormant cancer, as it has been shown that propranolol significantly inhibits processes associated with cancer growth [59]. It is also necessary to note that several studies have been published that did not find any effect of β blockers on cancer incidence, or even found a slight increase in cancer risk [60,61,62]. However, these studies usually did not differentiate between the effects of different β blockers and, therefore, propranolol’s effect on cancer incidence cannot be appropriately evaluated in these studies [63,64,65]. Moreover, another factor that might potentially influence the interpretation of data related to propranolol’s effect on cancer incidence might be the control group of subjects used. In the case that the control group of patients includes individuals with normal blood pressure, then the effect of propranolol on cancer incidence might be distorted, as it is known that hypertension itself can increase the risk of cancer [66,67,68].

Propranolol’s effect of reducing cancer incidence might be especially important for those individuals whose increased cancer risk results from long-term, exaggerated sympathoadrenal tone and consequently increased adrenergic signaling. For example, patients with polycystic ovary syndrome, obesity, some subtypes of hypertension, or those smoking combustible or electronic cigarettes containing nicotine represent individuals with increased cancer risks that might be related, at least partially, to increased activity of the sympathoadrenal system [6,69,70]. However, it is necessary to note that in individuals with exaggerated sympathoadrenal activity, the effect of propranolol might be less pronounced than in those with sympathoadrenal activity within the range of normal physiological values. Therefore, assessment of sympathoadrenal activity by determining heart rate variability may allow for a more appropriate assessment of propranolol’s effect on cancer incidence and progression.

Propranolol might also be potentially effective in cancer prevention for individuals accidentally exposed to carcinogens. However, further studies are necessary to assess whether propranolol might be used for cancer prevention. If this beneficial effect of propranolol is confirmed, then it will have several important consequences. For example, it will then be necessary to consider whether or not it is more appropriate to preferentially treat hypertensive patients with an increased cancer risk with propranolol, if possible.

### 2.2. Propranolol and Cancer Progression

Recently, propranolol has been routinely used for the treatment of problematic proliferating infantile hemangioma. Several mechanisms that could underlie propranolol’s effect on infantile hemangioma were proposed, including its inhibitory effect on angiogenesis, promotion of apoptosis in capillary endothelial cells, inhibition of vasodilatation and potentiation of vasoconstriction, and modulation of the renin-angiotensin system (for review, see [71]). However, even though infantile hemangioma is a benign tumor, some of the proposed mechanisms of action of propranolol might also be involved in inhibiting the progression of malignant tumors.

In support of this, findings from in vitro experiments and animal models of cancer have consistently demonstrated that propranolol inhibits cancer progression and the development of metastases [33,35,37,72,73,74,75,76,77]. Similarly, several retrospective clinical studies have shown a reduction in cancer proliferation and metastatic markers, as well as the prolonged survival of cancer patients, in those that were treated with propranolol for hypertension or other non-cancer diseases [44,50,51,78,79,80,81]. However, not all studies confirmed this beneficial effect of propranolol in cancer patients [60,82].

Importantly, a recent prospective study of propranolol as an off-label treatment has shown that patients treated with this β blocker have an 80% reduction in risk for melanoma recurrence [83]. Similarly, neoadjuvant propranolol treatment decreased the expression of the pro-proliferative Ki-67 and pro-survival Bcl-2 markers and increased pro-apoptotic p53 expression in a patient with stage III breast cancer [84]. Currently, several studies are ongoing to investigate the feasibility of combining propranolol with chemotherapy in cancer patients (e.g., [85]; for more, see ClinicalTrials.gov).

### 2.3. Propranolol and Efficiency of Conventional Anti-Cancer Treatment

Cancer diagnosis and treatment induces intensive and chronic stress, which is accompanied by increased adrenergic signaling in many patients [86,87]. Increased adrenergic tone could then reduce the effectiveness of cancer treatment. Importantly, besides its suppressive effect on cancer incidence and progression, propranolol might, via suppression of stress’s effect on cancer tissue, also significantly improve the efficiency of conventional oncological treatment modalities, such as surgery, chemotherapy, radiotherapy, and immunotherapy.

**Figure 2:**
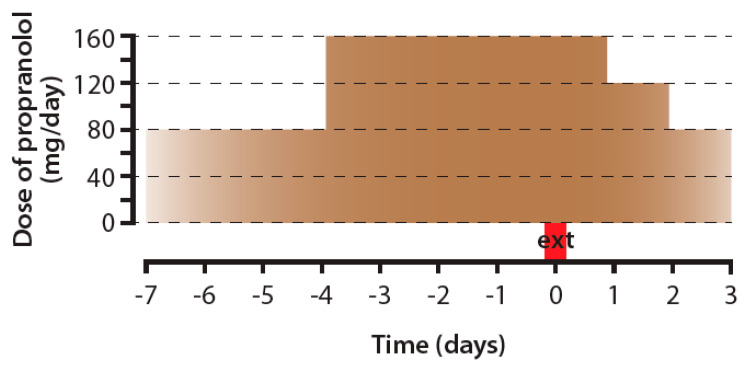
Dosage of propranolol used to prevent perioperative and postoperative metastases in a clinical study by Hiller, et al. [96]. At the beginning (day −7), patients received 40 mg of propranolol twice a day; from day −4, they received 80 mg twice a day, and after the operation, the dose was gradually reduced. ext—day of tumor tissue extirpation.

***Resection of primary cancer.*** Surgical removal of a tumor is one of the most commonly used therapeutic modalities in oncology. However, resection of the primary tumor is often accompanied by alteration of many biological processes, including exaggerated β-adrenergic signaling, which potentiates the development of metastases and therefore worsens the clinical course of cancer [88,89]. Animal experiments have shown that perioperative treatment by propranolol and a COX-2 inhibitor significantly reduced lung tumor retention in rats intravenously inoculated with MADB106 syngeneic MADB106 mammary adenocarcinoma cells [90,91]. Similarly, perioperative treatment with propranolol reduced the markers of metastasis and tumor burden in patients with breast, ovarian, and colorectal cancer [92,93,94,95,96,97] (Figure 2). Importantly, these clinical studies also proved the safety of propranolol use in cancer patients during the perioperative period.

***Chemotherapy.*** In vitro experiments have demonstrated that propranolol increases the efficacy of several chemotherapeutics used in oncology. For example, propranolol potentiated the anti-angiogenic and anti-tumoral efficacy of 5-fluorouracil and paclitaxel in a murine, orthotopic xenograft model of triple-negative breast cancer [98]. Propranolol also increased the in vitro and in vivo efficacy of sunitinib on mouse melanoma during increased adrenergic signaling [99]. Moreover, propranolol exerted a synergic effect with doxorubicin in inhibiting the viability of liposarcoma and leiomyosarcoma cells and increased the response to docetaxel in angiosarcoma and solitary fibrous tumor patient-derived cells. This effect of propranolol was mediated by a reduction in the activity of the multidrug resistance efflux pump P-gp, thereby increasing the intracellular doxorubicin concentration and its anti-tumor activity [100].

***Radiotherapy.*** Combined treatment with propranolol and dichloroacetate enhances the effects of chemoradiation and sensitizes head and neck squamous cell carcinoma to cisplatin and radiation during both in vitro and in vivo conditions [101]. Propranolol also increased the anti-tumor efficacy of radiation in CT26 murine colon adenocarcinoma. This effect was mediated by a reduction in tumor cell resistance to radiation-induced cell death and by the exaggeration of anti-tumor immunity [22].

***Immunotherapy.*** It has been shown that propranolol enhanced the efficacy of anti-(α)PD-1 checkpoint blockade in a murine model of melanoma [102]. Propranolol also increased the efficacy of tumor antigen lysate vaccine in mice with breast cancer induced by implantation of breast tumor pieces [103].

***Hematopoietic cell transplantation (HCT).*** Data indicate that propranolol might reduce the rate of tumor progression, not only in solid but also hematologic malignancies. This effect might be of importance, especially during the peri-transplant period characterized by increased activity of adrenergic signaling induced by psychosocial stress. Therefore, a proof-of-concept randomized pilot study assessed the feasibility of propranolol treatment in patients receiving an autologous HCT for multiple myeloma. The study showed that there was a good tolerance to propranolol, which was reflected by the high adherence rate and study retention [104].

### 2.4. The Issues Related to Propranolol Usage in Oncology

There are several factors that determine the effects of propranolol on processes related to cancer initiation, progression, and treatment. Published data indicate that propranolol’s effect on carcinogenesis and cancer progression is a complex phenomenon that could reflect its effects on normal cells and the tumor micro- and macroenvironment, as well as its vasodilatory effect (e.g., steal effect on tumor perfusion). However, these factors are only characterized in some types of cancer. Therefore, further studies are necessary to determine the efficacy of propranolol for the prevention and treatment of various types of cancer. Mentioned below are some of the factors affecting propranolol’s efficacy in cancer treatment and prevention.

***Cancer type.*** Adrenergic signaling, which is reduced by propranolol, does not play an equal role in all cancer types [105]. For example, available data indicate that, whereas gastric cancer is influenced mainly by acetylcholine released from parasympathetic nerves (cholinergic signaling) [106], prostate cancer is affected by both parasympathetic and sympathetic nerves depending on the stage of cancer [7], and breast cancer is affected almost exclusively by adrenergic signaling [107]. Therefore, propranolol might be preferentially used for the prevention and treatment of those cancers in which adrenergic signaling plays an important role, such as prostate and breast cancer.

***Dosage.*** It has been shown that propranolol’s inhibitory effect on tumor growth in a mouse model of melanoma depends on dosage and has a U shape [108]. In support of this, a U-shaped effect was also found in a retrospective study investigating the effect of antihypertensive treatment on breast cancer incidence in hypertensive women. In this study, it was found that the hazard ratio was lowest in patients treated with 25 to 50 mg of propranolol per day than in women treated with either higher or lower doses [109].

***Route of administration.*** In both animal experiments and clinical trials, propranolol is administered systemically, either by injection or orally. However, propranolol can also be administered locally. Topical administration of propranolol could potentially reduce the adverse effects accompanying systemic administration and also increase its concentration in cancer tissue. For example, topical propranolol application might be of interest in the treatment of melanoma, as several studies have shown its efficacy in the treatment of this cancer type [83,110].

***Combination with other drugs.*** The efficacy of propranolol might be increased by being given in combination with drugs that affect other aspects of cancer pathogenesis. For example, experimental data have shown that combining propranolol with 2-Deoxy-D-glucose efficiently prevented prostate cancer cell proliferation, induced cell apoptosis, altered mitochondrial morphology, inhibited mitochondrial bioenergetics, and aggravated endoplasmic reticulum stress in vitro, while also suppressing tumor growth in vivo [111]. Another example of increased propranolol efficacy is related to its effect on the metabolism of glucose in cancer cells. It was shown that propranolol, via inhibition of mitochondrial metabolism, exerts potent anti-cancer effects in head and neck squamous cell carcinoma, but also increases glycolytic activity of these cancer cells, which may limit its effectiveness. However dichloroacetate, a clinically available glycolytic inhibitor, enhanced this propranolol induced anti-cancer effect [101]. Another study demonstrated that combined treatment with propranolol and another six repurposed drugs might represent a new approach for treating metastatic breast cancer. Furthermore, it was found that this combination inhibited epithelial-to-mesenchymal transition and augmented capecitabine efficacy [112].

***Physical and psychosocial characteristics of patients.*** The effect of propranolol on cancer might be also determined by the health status of the patient. For example, in older patients with several comorbidities, propranolol can exert various side effects that might affect its effect on cancer as well as reduce compliance. Moreover, psychosocial factors might play a significant role as data from studies investigating the effect of psychotherapy, which also reduces adrenergic signaling, have shown that this approach is effective, especially in some subgroups of cancer patients [113]. Analogously, it can be expected that propranolol will be especially effective in individuals exposed to intensive and chronic stress, including newly diagnosed or socially isolated cancer patients.

***Metronomic chemotherapy.*** If the anti-cancer activity of propranolol is confirmed in clinical trials, the introduction of propranolol into conventional cancer treatment may allow oncologists to reduce the dosage of chemotherapy and also possibly radiotherapy and other treatments, thus reducing their adverse effects on the patient’s body.

## 3. Drugs Reducing Density or Activity of Nerves Innervating Cancer Tissue

It has been suggested that the innervation of cancer tissue is a new hallmark of cancer [114]. In support of this, it has been demonstrated that increased nerve size and density represents a negative prognostic marker of cancer disease [7,115,116,117,118].

There are several sources of nerves in cancer tissue, including nerves already present in tissue before the transformation of normal tissue cells into cancer, phenotypically transformed neurons that innervate the tissue of tumor origin [119], new branches of nerves growing to the tumor tissue from nerves localized around the tumor tissue, and axons of new neurons migrating from a distant part of the central or peripheral nervous system into the tumor tissue or its vicinity [120]. The main stimuli for the ingrowth of new nerves into cancer tissue are nerve growth factors released by cells in the tumor microenvironment [121].

**Figure 3:**
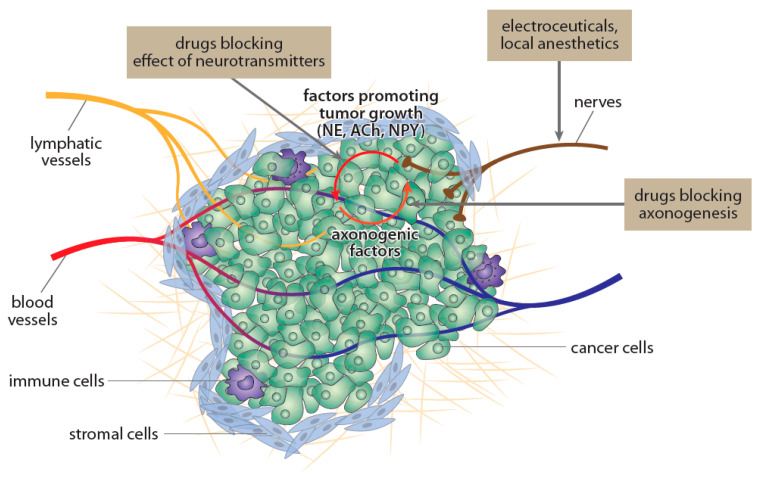
Schematic depiction of the vicious cycle created between nerves and cancer cells. Nerves innervating cancer tissue release neurotransmitters (e.g., acetylcholine (Ach), norepinephrine (NE), neuropeptide Y (NPY)) that stimulate tumor growth. The more cancer cells, the more released molecules, inducing the ingrowth of new axons (e.g., nerve growth factor, NGF). Therefore, administration of antibodies against NGF or suppression of the release of other molecules related to neoaxoneogenesis might be useful in reducing cancer growth. Modified according to Venkatesh and Monje [123].

Neurotransmitters released from nerves innervating cancer exert complex effects on cancer progression and the development of metastases [122]. These effects depend on the type of nerves [105] and their density in cancer tissue [7,115,116,117,118]. For example, norepinephrine released from sympathetic nerves plays an important role in the initial phases of prostate cancer development, while acetylcholine released from parasympathetic nerves potentiates the progression of prostate cancer and development of metastases [7]. Importantly, neurotransmitters such as norepinephrine, released from nerve endings in tumor tissue, increase cancer cell proliferation. Because cancer cells release nerve growth factors that potentiate the growth of new axons into cancer tissue, positive feedback is established. This vicious cycle might represent a new target in cancer treatment (Figure 3).

### 3.1. Drugs Reducing Nerve Growth Factor-Related Signaling

The effect of reducing cancer innervation by the administration of drugs that suppress neoaxonogenesis in tumor tissue has been investigated in both animal and human cancers. It has been shown that nerve growth factor (NGF) potentiates neoaxonogenesis and that systemic or local administration of NGF receptor inhibitors or antibodies against NGF reduces the density of nerves in cancer tissues, including breast and pancreas [23,124,125].

It has been suggested that antibodies to nerve growth factors (e.g., tanezumab) [126], or drugs blocking the release of exosomes (such as those containing the axonal guidance molecule, EphrinB1) [127] into cancer tissue, might be used for the treatment of cancer in humans.

### 3.2. Botulotoxin

Botulotoxin inhibits the release of acetylcholine from the nerve endings of motor and parasympathetic nerves. As it has been demonstrated that parasympathetic nerves exert a stimulating effect on the development and progression of some cancers [7,106], the effect of botulinum toxin on cancer was investigated in animal models, as well as in cancer patients. Botulotoxin treatment reduced tumor size and increased the apoptotic rate of pancreatic cancer in athymic nude mice [128], while, in humans, botulotoxin treatment resulted in increased apoptosis of cancer cells in the side of the prostate injected with botulotoxin [129].

### 3.3. Electroceuticals

In 2013, Famm, et al. [130] introduced the term “electroceuticals” into the scientific literature for devices that might treat diseases or reduce their symptoms by modulation of neural activity. These devices can be surgically implanted or attached to the skin and affect the transmission of action potentials in certain nerves via the generation of electrical impulses with programable intensity, frequency, and shape. Electroceuticals are now approved for the treatment of epilepsy and depression [131]. However, clinical studies indicate that electroceuticals might be effective in the treatment of a much wider spectrum of diseases, including pain, sepsis, lung injury, rheumatoid arthritis, diabetes, gastrointestinal diseases, and many others [131,132,133].

Electroceuticals are used also in cancer patients for the reduction of chemotherapy-induced nausea and vomiting [134]. Recently, its use in more directed cancer treatment was proposed [135,136]. It can be hypothesized that, in some circumstances (e.g., inoperable cancer), it will be possible to modulate the transmission of signals from nerves to cancer by electroceuticals, thereby reducing the stimulatory effect of the nervous system on cancer growth.

It is necessary to note that the term electroceuticals in some papers is also used to describe drugs that affect the electrical properties of cells, including drugs affecting ion channels [137]. Therefore, electroceuticals, in general, might include also local anesthetics and other drugs altering the transmission of action potentials. These drugs might be also used for the treatment of cancer as they block the transmission of signals from the nervous system to effector cells in the tumor microenvironment.

### 3.4. Local Anesthetics

Signals, in the form of action potentials transmitted along the axons of autonomic and sensory neurons, participate in the modulation of cancer growth, as well as in the modulation of nervous system function by cancer. Besides electrical signals transmitted along axons, it has been shown that cancer cells may develop neurite-like protrusions that form contact with nerve endings [121], and that voltage-gated sodium channels play a role in cancer cell invasion and metastasis [138,139]. Therefore, it might be hypothesized that modulation of the electrical properties of cancer tissue and its vicinity might affect its progression.

The transmission of signals between the nervous system and cancer tissue, along with the electrical activity of cancer tissue, might be reduced by application of local anesthetics. Local anesthetics in the form of gels might be useful for interrupting signals conducted by superficial nerves innervating cancer localized in the skin. Therefore, these drugs might potentially reduce the stimulatory effect of nerves innervating melanoma. However, even though several papers have shown that local anesthetics might exert direct anti-cancer effects [140,141,142], there are no data in the available literature showing that local anesthetics can be used to suppress cancer via the attenuation of signal transmission between the nervous system and cancer tissue. Therefore, further research is necessary to assess whether blocking the transmission of action potentials via local anesthetic in axons innervating cancer tissue might affect cancer progression. The most plausible use of local anesthetics would be in the treatment of superficial cancers, such as melanoma.

## 4. Drugs Interfering with Cancer Effects on the Brain

Cancer might affect several brain functions, including homeostatic regulation of energy balance, cognition, mood, and sleep. Importantly, alterations of brain functions might, at least partially, also affect the course of cancer disease and therefore the quality of life and survival of cancer patients. Therefore, attenuation of cancer’s effect on the brain might represent a new therapeutic target in oncology.

**Figure 4:**
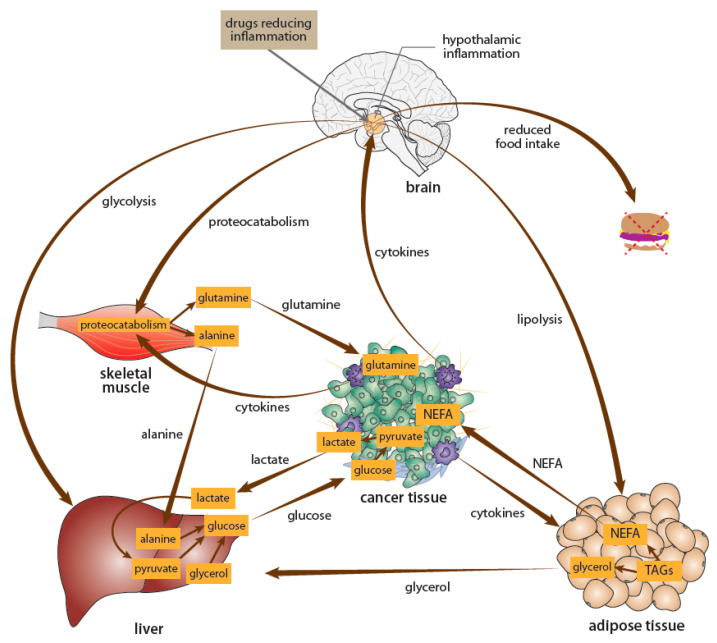
Schematic depiction of the mechanisms involved in the development of cancer cachexia. In addition to peripheral mechanisms, hypothalamic inflammation induced by cytokines and other factors synthesized in the tumor microenvironment and peripheral tissues is involved in the development of cachexia. Hypothalamic inflammation disrupts regulation of food intake and increases energy expenditure. Therefore, drugs such as aspirin or metformin might directly or indirectly reduce hypothalamic inflammation and could be useful in the treatment of cancer anorexia and cachexia. Modified according to Argiles, et al. [149]. NEFA—non-essential fatty acids; TAGs—triacylglycerols.

***Anorexia and cachexia.*** Cancer-induced alteration of specific brain circuits might significantly affect energy balance (Figure 4). In particular, signaling molecules released from the tumor microenvironment (e.g., cytokines) might induce neuroinflammation in the hypothalamus, the main brain center maintaining energy homeostasis [143]. This hypothalamic neuroinflammation might subsequently disrupt the precise regulation of metabolic processes in the body and thus contribute to the progression of anorexia and cachexia in individuals with cancer [144]. Hypothalamic neuroinflammation is activated by several factors, including pro-inflammatory molecules, and is maintained by activation of NF-κB gene expression in hypothalamic neurons and glia cells [145].

Several approaches might be used to attenuate hypothalamic inflammation in cancer patients with the goal of attenuating the development of anorexia and cachexia. These might include the reduction of cytokine synthesis in the periphery, as well as in the brain, by drugs already approved for the treatment of non-cancerous diseases, including aspirin and metformin. In addition, hypothalamic inflammation might also be reduced by dietary supplements such as polyunsaturated fatty acids [146] or by regular physical activity [147]. In addition, drugs interfering with brain signaling mediated by neuropeptides such as neuropeptide Y or agouti-related protein might restore the functions of the neuronal circuits regulating energy balance [148].

### 4.1. Aspirin

One of the main mechanisms of action of aspirin is mediated by the suppression of cyclooxygenase activity, followed by the reduction of prostaglandin synthesis [150]. Importantly, it was shown that aspirin administration resulted in a partial recovery of body weight and food intake in Walker 256 tumor-bearing rats [151]. Even if the mechanism responsible for this effect was not investigated further, based on the known suppressive effect of aspirin on prostaglandin synthesis in the hypothalamus [152], it can be hypothesized that the potential anorexia- and cachexia-reducing effect of aspirin might be, at least partially, mediated by the reduction of hypothalamic inflammation. This hypothesis is further supported by data showing that aspirin modulates the activity of the adenosine monophosphate (AMP)-activated protein kinase (AMPK) signaling pathway that plays a central role in the regulation of energy balance by the hypothalamus [153].

### 4.2. Metformin

Metformin exerts complex molecular effects, including the activation of adenosine monophosphate (AMP)-activated protein kinase (AMPK) signaling [154], and inhibits a NF-κB pro-inflammatory pathway [155] in the periphery, as well as in the brain. Data indicate that this drug might also be repurposed for cancer prevention and treatment [156]. Based on the anti-inflammatory and energy homeostasis-maintaining effects of metformin, it can be hypothesized that this drug might also be used for suppressing hypothalamic inflammation and therefore the reduction of anorexia and cachexia development in cancer patients. In support of this, it was shown that metformin exerts an anti-cachexic effect in a murine B16-F1 cell line induced cancer cachexia model [157].

## 5. Conclusions

The neurobiology of cancer has revealed new mechanisms and pathways participating in cancer initiation and progression. These cancer-related mechanisms and pathways might represent a target for new drugs, as well as for drugs already approved for the treatment of non-cancer diseases. Currently, several clinical trials are ongoing to investigate the effect of drugs such as propranolol or metformin in the treatment of human cancer. Importantly, the repurposing of these approved drugs might significantly speed up their introduction into the cancer treatment regimen. However, it is necessary to note that several questions need to be answered before introducing these drugs into conventional oncological treatment. Therefore, further studies will be necessary to evaluate against which cancer types these drugs are the most efficient, determine the optimal therapeutic dosage of these drugs, their potential adverse effects depending on interaction with other drugs used by patients, and other factors.
